# Copper Perchlorate Hexahydrate: An Efficient Catalyst for the Green Synthesis of Polyhydroquinolines under Ultrasonication

**DOI:** 10.5402/2011/948685

**Published:** 2011-04-27

**Authors:** Saurabh Puri, Balbir Kaur, Anupama Parmar, Harish Kumar

**Affiliations:** ^1^Department of Chemistry, Punjabi University, Patiala 147002, India; ^2^Post Graduate Department of Chemistry, M.M. Modi College Patiala, Patiala 147001, India; ^3^Department of Chemistry, Sant Longowal Institute of Engineering and Technology, Longowal 148106, India

## Abstract

Copper perchlorate hexahydrate as an efficient catalyst was used for the synthesis of polyhydroquinolines by four-component condensation reaction of aldehyde, ethyl acetoacetate, dimedone, and ammonium acetate in excellent yields and short reaction times at room temperature under ultrasound irradiation. This novel synthetic method is especially favoured because it provides a synergy between copper perchlorate hexahydrate and ultrasound irradiation which offers the advantages of high yields, short reaction times, simplicity, and easy workup compared to the conventional methods reported in the literature.

## 1. Introduction

Multicomponent reactions (MCRs) have emerged as an efficient and dominant tool in modern synthetic organic chemistry allowing the facile creation of several new bonds in a one-pot reaction. Clearly for multistep synthetic procedures, the number of reactions and purification steps is among the most significant criteria for the efficiency and feasibility of the process and should be as low as possible. Therefore research in academia and industry has increasingly emphasized the use of MCRs as well as domino reaction sequences for a broad range of products [1, 2]. Ultrasound is widely used for improving the traditional reactions that use expensive reagents, strongly acidic conditions, long reaction times, and high temperatures unsatisfactory yields [3]. 4-substituted 1,4-dihydropyridines (1,4-DHPs) are well known as Ca^2+^ channel blockers and emerged as one of the most important classes of drugs for the treatment of cardiovascular diseases, including hypertension [4, 5]. In view of the importance of polyhydroquinoline derivatives, many classical methods for their synthesis were reported [6–11] using conventional heating and refluxing approaches in the presence of an organic solvent. These methods, however, involve long reaction times, harsh reaction conditions, and the use of a large quantity of volatile organic solvents and generally lead to low yields. Therefore, it is necessary to develop an efficient and versatile method for the preparation of 1,4-dihydropyridines, and the progress in this field has been recently remarkable including the promotion of microwave [12], TMSCl [13], ionic liquids [14, 15], polymers [16, 17], and Yb(OTf)_3_ [18]. The multicomponent reactions are powerful tools in the modern drug discovery process and allow fast, automated, and high-throughput generation of organic compounds [19]. The possibility of performing multicomponent reactions under solvent-free conditions with a heterogeneous catalyst could enhance their efficiency from an economic as well as an ecological point of view. In recent years heterogeneous catalysts have been gaining more importance due to environmental-economic factors. The catalyst is generally of low cost and can be easily handled or removed. Herein, we would like to report an efficient and greener route for the synthesis of polyhydroquinolines from the reaction of readily available and nonexpensive starting materials (dimedone, aromatic aldehydes, ethyl acetoacetate, ammonium acetate, and copper perchlorate hexahydrate) under solvent-free conditions using ultrasonic irradiation. 

## 2. Experimental

### 2.1. Chemicals and Apparatus

Liquid carbonyl compounds were purified by distillation before use. All melting points recorded are uncorrected, open capillary measurements, using sulphuric acid bath. NMR spectra were recorded on AL-300F (Bruker) FT NMR spectrophotometer using tetramethylsilane (TMS) as internal standard. All solvents were reagent grade and used as received. The reactions were performed in open vessels. 

### 2.2. General Procedure for the Synthesis of Polyhydroquinoline Derivatives

A mixture of aryl aldehyde (1 mmol), 5,5-dimethyl-1,3-cyclohexanedione (1 mmol), ethyl acetoacetate (1 mmol) and ammonium acetate (1 mmol) was added to Cu(ClO_4_)_2_·6H_2_O (15 mol%), and the reaction mixture was exposed to ultrasound irradiation 20–40 min (completion of the reactions was monitored by TLC). After the completion of reaction, the reaction mixture was diluted with ethyl alcohol and stirred for 10 minutes at 80°C. The residue was filtered hot and kept at room temperature, and the resulting crystalline product was collected by filtration. The product formed was recrystallized from ethanol. The formation of products was confirmed by comparing the melting points and NMR data with authentic samples and literature data.

### 2.3. Selected Spectral Data of the Selected Products

**2,7,7-Trimethyl-5-oxo-4-phenyl-1,4,5,6,7,8-hexahydroquinoline-3-carboxylic acid ethyl ester (1a)**

mp 202–204°C. IR (KBr) cm^−1^: 3287, 3077, 2964, 1696, 1610. ^1^H-NMR (CDCl_3_) *δ*: 0.93 (3H, s), 1.06 (3H, s), 1.20 (3H, t, *J *= 7.1 Hz), 2.12–2.28 (4H, m), 2.34 (3H, s), 4.05 (2H, q, *J *= 7.1 Hz), 5.06 (1H, s), 6.63 (1H, s), 7.07–7.12 (1H, m), 7.17–7.22 (2H, m), 7.27–7.32 (2H, m).

**4-(4-Methoxyphenyl)-2,7,7-trimethyl-5-oxo-1,4,5,6,7,8-hexahydroquinoline-3-carboxylic acid ethyl ester (1b)**

mp 257-258°C. IR (KBr) cm^−1^: 3276, 2956, 1703, 1648, 1606, 1496, 1381, 1215, 1031, 765. ^1^H-NMR (CDCl_3_ + DMSO-d_6_) *δ*: *δ* = 0.95 (3H, s), 1.09 (3H, s), 1.21 (3H, t, *J *= 7.2 Hz), 2.01–2.10 (4H, m), 2.30 (3H, s), 3.70 (3H, s), 4.00 (2H, q, *J *= 7.2 Hz), 4.80 (1H, s), 6.65 (2H, d, *J *= 7.3 Hz), 7.10 (2H, d, *J *= 7.3 Hz), 8.65 (1H, s).

**4-(4-Fluorophenyl)-2,7,7-trimethyl-5-oxo-1,4,5,6,7,8-hexahydroquinoline-3-carboxylic acid ethyl ester (1f)**

mp 184-185°C. IR (KBr) cm^−1^: 3292, 2959, 1696, 1649, 1608, 1487, 1380, 1219, 1025, 764. ^1^H-NMR (CDCl_3_) *δ*: 0.92 (3H, s), 1.07 (3H, s), 1.18 (3H, t, *J *= 7.3 Hz), 2.13–2.25 (4H, m), 2.38 (3H, s), 4.05 (2H, q, *J *= 7.33 Hz), 5.02 (1H, s), 5.8 (1H, s), 6.85–6.89 (2H, m), 7.23–7.27 (2H, m).

**4-(2-Chlorophenyl)-2,7,7-trimethyl-5-oxo-1,4,5,6,7,8-hexahydroquinoline-3-carboxylic acid ethyl ester (1k)**

mp 209-210°C. IR (KBr) cm^−1^: 3063, 2956, 1721, 1640, 1611, 1467, 1384, 1227, 1021, 745. ^1^H-NMR (CDCl_3_ + DMSO-d_6_) *δ*: *δ* = 0.95 (3H, s), 1.05 (3H, s), 1.20 (3H, t, *J *= 7.2 Hz), 2.01–2.21 (4H, m), 2.40 (3H, s), 4.05 (2H, q, *J *= 7.2 Hz), 4.60 (1H, s), 7.10–7.30 (4H, m), 7.60 (1H, s).

## 3. Results and Discussion

**Scheme 1:**
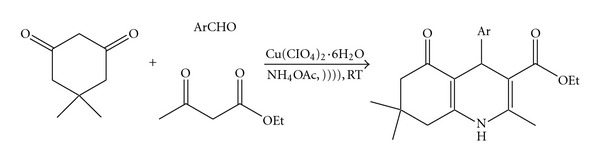
Solvent-free synthesis of polyhydroquinolines 1a–1l under ultrasound irradiation.

In continuation of our work to develop new and ecofriendly synthetic methodologies [20–23], herein, we report an expedient protocol for the synthesis of polyhydroquinolines. (Scheme 1). The reactions were carried out at room temperature for 20–40 min in the presence of 15 mol% of the copper perchlorate hexahydrate at 35 kHz under ultrasound irradiation. To determine the amount of the catalyst in this reaction, benzaldehyde was used as one of the reactants for 20 min under ultrasound irradiation in the presence of varying mol% of the copper perchlorate hexahydrate separately. The best results were obtained using 15 mol% of the catalyst. Using lesser amount of the catalyst resulted in lower yields, while higher amount of the catalyst did not affect reaction times and yields. In the absence of the catalyst, the yield was found to be very low. To verify the effect of ultrasound irradiation on this procedure, the synthesis of polyhydroquinolines was done in the presence of varying mol% of the copper perchlorate hexahydrate with and without ultrasound irradiation (Table 1). In all reactions, it was found that the use of ultrasound leads to faster reaction and higher yields. So it shows that use of the ultrasound improves the rate of reaction and also yields of products formed. A wide range of substituted aldehydes were used to give excellent yield of products (1a–1l) (Table 2). Reactions were also performed using ethanol as a solvent, but the yield was less than the in solvent-free conditions.

## 4. Conclusion

We have introduced a new and highly efficient catalyst, copper perchlorate hexahydrate, for the synthesis of polyhydroquinolines. The significant aspects of our methodology are efficiency, generality, high yield, short reaction time, low cost, cleaner greener reaction profile, ease of product isolation, the use of alternative source of energy, and ultimately conformity with the green chemistry protocols.

## Figures and Tables

**Table 1:** Effect of amounts of catalyst, copper perchlorate hexahydrate with or without sonication for synthesis of 2,7,7-Trimethyl-5-oxo-4-phenyl-1,4,5,6,7,8-hexahydroquinoline-3-carboxylic acid ethyl ester.

Entry	Cu(ClO_4_)_2_·6H_2_O mol%	With sonication		Without sonication	
		Yield (%)	Time (min)	Yield (%)	Time (min)
(1)	0	Nil	90	Nil	90
(2)	5	24	60	10	70
(3)	10	57	40	23	55
(4)	15	90	20	33	40
(5)	20	90	20	33	40

**Table 2:** Copper perchlorate hexahydrate catalyzed solvent-free synthesis of polyhydroquinolines 1a–1l under ultrasound irradiation.

Entry	Products	Ar	Time (min)	Yield (%)	mp °C (Lit mp °C)
(1)	1a	C_6_H_5_	20	96	203-204 (202–204)^16^
(2)	1b	4-MeOC_6_H_4_	25	97	257-258 (257–259)^16^
(3)	1c	4-ClC_6_H_4_	25	95	244-245 (245-246)^12^
(4)	1d	4-NO_2_C_6_H_4_	35	88	242-243 (242–244)^12^
(5)	1e	3-NO_2_C_6_H_4_	35	86	176-177 (177-178)^5^
(6)	1f	4-FC_6_H_4_	25	94	184-185 (184–186)^16^
(7)	1g	4-OHC_6_H_4_	40	89	232-233 (232–234)^16^
(8)	1h	2-NO_2_C_6_H_4_	25	90	207-208 (206–208)^5^
(9)	1i	4-BrC_6_H_4_	30	91	254-255 (253–255)^16^
(10)	1j	3-ClC_6_H_4_	25	93	231-232 (230–232)^22^
(11)	1k	2-ClC_6_H_4_	25	93	209-210 (208–210)^12^
(12)	1l	4-MeC_6_H_4_	25	93	260-261 (260-261)^16^
